# Systematic Review and Meta-Analysis on Angiotensin Converting Enzyme 2 in Head and Neck Region

**DOI:** 10.7759/cureus.33673

**Published:** 2023-01-11

**Authors:** Shivani Sivasakthivel, Pratibha Ramani, Reshma Poothakulath Krishnan

**Affiliations:** 1 Department of Oral and Maxillofacial Pathology, Saveetha Dental College and Hospitals, Chennai, IND

**Keywords:** oral squamous cell carcinoma, head and neck neoplasms, head and neck, sars cov 2, angiotensin converting enzyme 2, ace2

## Abstract

The objective of this systematic review was to investigate the expression of angiotensin converting enzyme 2 (ACE 2) in the head and neck region. We examined the evidence of the association of ACE 2 expression in oral tissues, salivary glands, and head and neck carcinoma. We searched Pub Med/Medline, Biorxiv, and Google Scholar to identify relevant literature. Studies reporting ACE 2 expression in human oral tissues and with a focus on head and neck carcinoma samples were included. From 110 studies, we extracted 15 studies analyzing the distribution and expression of ACE 2 in different head and neck tissues - olfactory mucosa and nasopharynx n=5, oral mucosa n=5, salivary gland n=5, head and neck squamous cell carcinoma patients n=3.

ACE 2 was found to be expressed at a 4.43-fold increase in the head and neck region (OR, 4.43; 95% CI, 3.76-5.22; I_2_= 97%, P_h_=<0.00001) when compared with controls (other tissues except for head and neck region). RNA expression of ACE 2 was 60% higher in head and neck squamous cell carcinoma patients than that in the normal tissues (OR=0.60, 95% CI, 0.04-9.26, P_h_=0.00001). In conclusion, the meta-analysis of the studies indicated that ACE 2 is highly expressed in olfactory mucosa, nasopharynx, oral mucosa, and salivary glands. Furthermore, the results indicate that ACE 2 expression is increased in patients with head and neck cancer.

## Introduction and background

ACE 2, a homolog of angiotensin converting enzyme (ACE), is known to be of pathophysiological importance in various human tissues in both healthy and disease states [1]. Since 2019, the focus of attention is towards “ACE 2” - a key cell receptor for the SARS-CoV-2 virus and its emerging variants. ACE 2 being the functional receptor of SARS-CoV-2 has become the potential risk site for infection and pathogenesis. As a transmembrane protein, ACE 2 also serves as an entry receptor for other viruses including, SARS-CoV and HCoV-NL63. ACE 2 is the counter-modulator component of the Renin-Angiotensin-Aldosterone System (RAAS) [2-5]. The renin-angiotensin system is the critical regulator of blood volume, systemic vascular resistance, and cardiorenal function.

Studies have demonstrated the importance of ACE 2 in maintaining the balance of RAAS. Considering that SARS-CoV-2 cell entry depends on the expression of ACE 2 entry genes, we could speculate that the transmissibility and clinical manifestations of SARS-CoV-2 could be affected by the levels of ACE 2 expression on the cell surface. Multiple physiologic roles are known for ACE 2 impacting systems but perhaps most notably related to SARS-CoV-2, pulmonary. ACE 2 has been described to limit severe acute lung injury. Expression of ACE 2 is different in varied organs and their tissues. It is important to understand the distribution of ACE 2 in cells in different parts of the epithelium but also between cell-bound. In most studies, ACE 2 expressions in normal tissues were analyzed and compared to differential expression in cancer types. ACE 2 is highly expressed in alveolar epithelial cells of the lungs, myocardial cells, renal tubular cells, endothelial cells, enterocytes, cholangiocytes, and bladder urothelial cells. A study by Xu et al. [6] in the year 2020 showed that in the head and neck region, oral tissues, especially epithelial cells of the tongue, express higher ACE 2 levels. The pervasive expression of ACE 2 points us in the direction of hypothesizing its physiological plot in many organs and tissues.

Though ACE 2 is physiologically protective, it is virally conducive. The head and neck sphere at the forefront, assessing ACE 2 expression at the head and neck region, is mattering much for determining the vulnerability of these anatomical sites. Accordingly, this review focuses on ACE 2 expression in the head and neck region.

## Review

Methods

The methodology of Preferred Reporting Items for Systematic Review and Meta-analysis (PRISMA) guidelines was followed. The criteria for considering studies for the systematic review were based on the PICOTS framework: Population, Intervention, Comparison, Outcomes, Timing, and Setting framework.

Search strategy

A systematic search was performed using PubMed/Medline, BioRxiv, and Google Scholar to identify relevant literature. The searches were limited to articles published since 1990. The electronic searches were supplemented with a manual search of citations from key review articles. The articles with database abstracts and available full texts, implying they met the criteria, were screened. The following search terms are useful:

(angiotensin converting enzyme 2) OR (ACE2) OR (ACEH) OR (Renin Angiotensin System) OR (Angiotensin I converting Enzyme 2) AND (COVID-19) OR (Coronavirus) AND (head and neck) AND (oral cavity) OR (mouth) AND (saliva) OR (salivary gland) AND (oropharynx) AND (nasopharynx) OR (nasal cavity) AND (tongue) AND (buccal mucosa) AND (cancer) OR (carcinoma) AND (oral squamous cell carcinoma) AND (oral potentially malignant disorders) AND (thyroid) AND (single cell RNA sequencing) AND (immunohistochemistry) were used.

 

Type of studies

The review included studies on human tissues. The searches were limited to English articles. Preprint articles were also taken into consideration derived over a few months. Studies assessing ACE 2 expression by RNA sequencing profiling, immunohistochemistry, immunoblotting, in situ hybridization, and reverse transcription-polymerase chain reaction (RT-PCR) were included.

Participants and inclusion criteria

Papers were included in the review if they had clearly reported the expression of ACE 2 in the head and neck sites in human subjects. Animal studies, pilot studies, and systematic reviews were not included in the review.

Literature search and study characteristics

**Figure 1:**
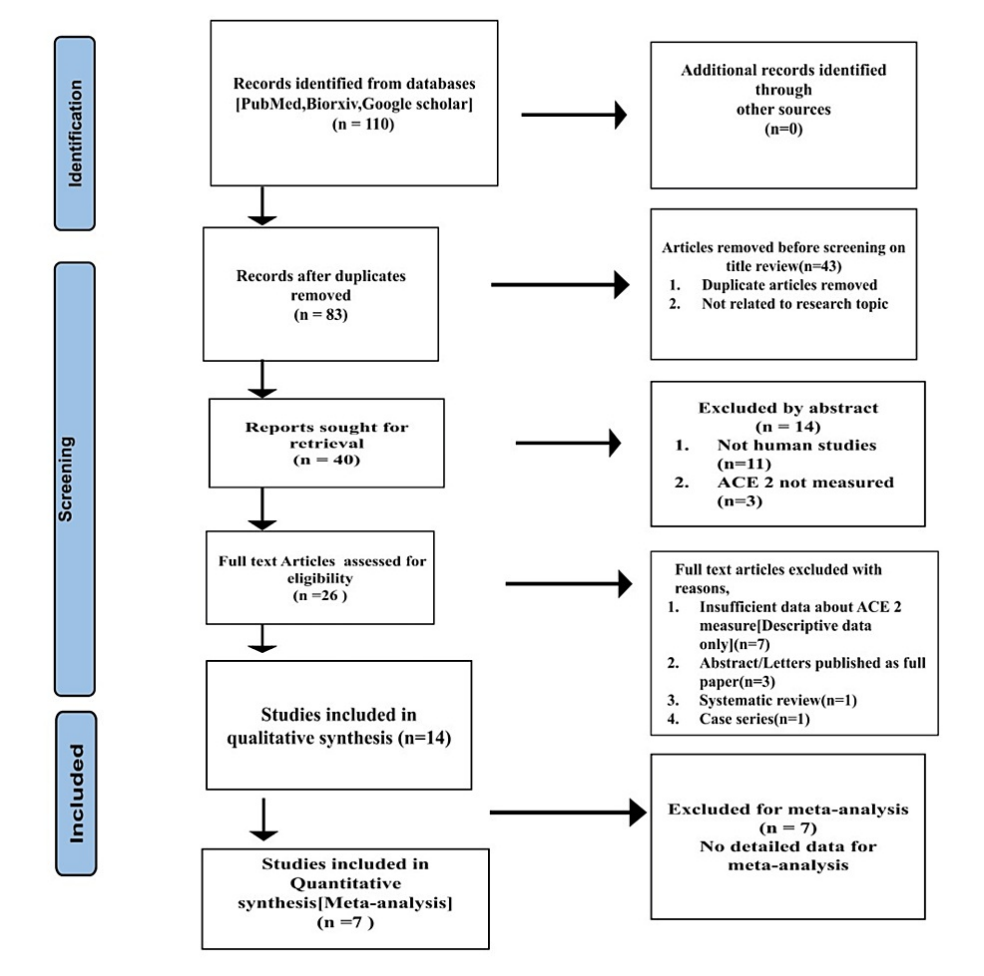
PRISMA study selection flow diagram ACE 2: angiotensin converting enzyme 2

The detailed steps of the systematized search and selection are shown in the PRISMA Study Selection Flow Diagram of the literature search process (Figure 1).

We extracted 14 studies from 110 studies (excluding duplicates and studies that did not meet our selection criteria) for qualitative analysis. Fourteen studies analyzed ACE 2 expression in public databases (human tissue datasets) on bioinformatic analysis. Four database studies pertained to head and neck carcinoma patients (Oral Squamous Cell Carcinoma n=1, Head and Neck Squamous Cell Carcinoma n=3). One study explored smoking-mediated ACE 2 expression. The characteristics of the study include anatomical districts (in the head and neck region), types of cells, and methods used for analyzing ACE 2 expression.

Intervention

As the rationale of this review is to collect evidence of the ACE 2 expression in the head and neck region, we did not look into an intervention in the human samples.

Outcome measure

The outcome measure for this review was the expression levels of ACE 2 in the head and neck region.

Statistical analysis

The statistical analysis was done using Revman Manager 5 (software version 5.2). Heterogeneity was estimated together with the I_2_ test. Separate subgroup analyses of the studies that assessed ACE 2 in the head and neck tissues and head and neck carcinoma were assessed. We conducted a random effect meta-analysis and estimated pooled OR (Odds Ratio) along with their 95% CI (Confidence Interval). Heterogeneity was estimated together with the I_2_ test. All the studies that were included in the review were not counted in the meta-analysis. Studies that did not report the OR (n=7) for ACE 2 expression in the head and neck region were excluded.

Data synthesis

When valid and relevant data were collected, we undertook a meta-analysis of the data. We grouped and analyzed studies based on the expression of ACE 2 in the normal head and neck tissues and carcinoma tissues. We conducted meta-analyzes in Review Manager software, using the Mantel-Haenszel method. Overall analysis was conducted with a fixed effect model.

Results

Of the 110 unique citations screened, we identified 14 studies relevant to ACE 2 expression in olfactory mucosa, nasopharynx, and oral mucosa n = 5, salivary gland n = 5, in head and neck carcinoma patients n = 4. Several included studies were relevant to more than one outcome of interest. Out of 14 studies, seven (n=7) studies met the criteria for meta-analysis.

Subgroup analysis

Subgroup analysis was conducted for ACE 2 expression in the head and neck tissues and in carcinoma patients separately.

Summarizing findings

We constructed a summary of findings for each included study to produce our review outcomes in Table 1.

**Table 1:** Characteristics of the included studies ACE 2 - angiotensin converting enzyme 2; sc RNA seq - single cell RNA sequencing; FANTOM 5 CAGE database - Functional ANnoTation Of the Mammalian genome project CAGE measures RNA expression and maps TSS in promoters; GTEx database - Gene type Tissue Expression; TCGA - tissue cancer genome atlas portal; TMPRSS 2 -transmembrane protease serine 2; FDR - false discovery rate; SARS CoV 2 - severe acute respiratory syndrome coronavirus 2 (Xu H et al. [4], Xu J et al. [6], Sakaguchi et al. [7], Sugnak et al. [8], Descamps et al. [9], Song et al. [10], Hamming et al. [11], Chen et al. [12], Sacconi et al. [13], Dai et al. [14], Han et al. [15], Chakladar et al. [16], Singh et al. [17], Raivoli et al. [18])

S. No	Author, year	Study type	Sample and data collection	Sample size	ACE 2 expression in the head and neck region	Methods used for detecting ACE 2 expression	Statistical analysis	Significant p value	P value	Results	Conclusion	Limitations
1	Xu H et al. 2020 [4]	Cohort study Bioinformatic analysis	Datasets from Tissue Cancer Genome Atlas Portal (TCGA) containing 13 organ types with Para- carcinoma normal tissues. Database of 14 organ types with normal tissues from FANTOM5 CAGE Control database n=695 Para-carcinoma tissues	Oral cavity [n=32]:Tongue n=13, Base of tongue n=2, Floor of mouth n=3, n=14 tissue into the category of oral cavity. In-house cohort n=4	Oral Epithelial cells	RNA sequencing profiling	t test was performed to compare the ACE 2 expression	p <0.05 was considered to be significant	Mean ACE 2 expression of tongue n=13 together oral site tissues n=19 p =0.062	In oral cavity, # 0.52% of ACE 2 positive cells in oral tissues # 95.86% ACE 2 positive cells is seen in tongue tissues # 93.38% ACE 2 positive cells is seen in epithelial cells # <0.5% of T Cells shows ACE 2 expression # 0.5% of B Cells shows ACE 2 expression # <0.5% of Fibroblasts shows ACE 2 expression	ACE 2 was higher in oral tongue than buccal and gingival tissues. ACE 2 positive cells of oral tissues includes epithelial cells, T cells, B cells, fibroblasts and higher expression was seen in epithelial cells that too in tongue epithelial cells.	Due to the limitation of the sample size, p value [p value = 0.062] was not significant.
2	Xu J et al. 2020 [6]	Observational study Bioinformatic analysis	Bulk tissue RNA seq data samples from TCGA, used for ACE 2 analysis included Head and Neck cancer Squamous cell carcinoma patients Single cell RNA seq Oral Cancer dataset were downloaded from the Gene Expression Ominous (GEO)	Normal controls-44 Oral cancer samples-502	Head and neck squamous cell carcinoma patients	scRNA seq (single cell RNA sequencing) profiling Bulk tissue RNA seq profiling	All analyses were performed in R (R version 3.6.1) and GraphPad 8.	P-value ≤ 0.05 was considered to be significant	Mean expression of ACE 2 in Oral cancer group- 7.363with SD- 1.843 Mean expression of ACE 2 in control group- 7.714 with SD- 1.406 ACE 2 expression in older head and neck squamous cell carcinoma patients is higher than the younger group with p=0.023 and the female group with p=0.801	ACE 2 expression was higher in tumor cells compared to normal control (NC) tissues	In digestive tract organs, ACE 2 expression gradually increased from the oral cavity to the oesophagus, stomach and the colon. ACE 2 expression in the older group was higher compared to the young group. In oral cancer patients, ACE 2 expression was significantly increased in the female patients. ACE 2 expression was higher in tumor cells compared to normal tissues. ACE 2 expression in the tissues are gradually increased from inflammation to metaplasia and then to cancer.	ACE 2 expression in histological grading of carcinomas to be correlated.
3	Sakaguchi et al. 2020 [7]	Case control study	Formalin-fixed paraffin-embedded blocks of pathological samples from dental hospital	15 tongue samples were mainly diagnosed with hemangioma of the dorsal tongue, no dysplastic changes, inflammation, or fungal infection was detected. The 16 gingival samples included the sulcular and gingival epithelium, and these samples had mild to severe inflammation due to epulis. Case:31 Control: Submandibular gland samples-positive control, Lymph node- negative control 5 patients with squamous cell carcinoma were used as controls	Gingival tissues Submandibular glands Tongue Taste buds	Immunohistochemistry	Levels of staining were graded as: no staining; definite staining of some cells, i.e., <30%of cells; definite staining of a majority of cells, i.e., <70%of cells. Counting method reported by Lucas with some modifications were used for cell counting.	p value not mentioned	p value not mentioned. For 15 tongue tissues, the patients were 10– 80 years of age, with a mean of 53.6 years. Six of the samples were from men, and nine from women. For 16 gingival tissues, the patients were 20– 91 years of age, with a mean of 51.88 years. Six of the samples were from men, and ten from women.	Tongue tissues: No staining Surface layer - 46.7% (7/15) Horny layer- 13.3% (2/15) Definite staining of some cells, i.e., <30% of cells Surface layer-40.0% (6/15) Horny layer-73.3% (11/15) Spinous–Basal Cell Layer - 60.0% (9/15). Definite staining of a majority of cells, i.e., <70% of cells Surface layer - 13.3%(2/15) Horny layer - 13.3%(2/15) Spinous–Basal Cell Layer - 40.0% (6/15) Gingival tissues: No staining Surface layer - 100.0% (16/16) Horny layer- 68.8 % (11/16) Definite staining of some cells, i.e., <30% of Cells Horny layer-25.0% (4/16) Definite staining of a majority of cells, i.e., <70% of cells Horny layer- 6.3% (1/16)	ACE 2 expression was mainly observed in the nuclei and cytoplasm of the spinous and basal cell layers. Consistent expression of ACE 2 protein in the epithelial layer of taste buds. Nucleus and cytoplasm of spinous basal cell layer in gingival squamous epithelium express ACE 2. Serous cells, ductal epithelium of salivary gland positively stained for ACE 2.	Limited sample size Unmatched control and control size.
4	Sugnak et al. 2020 [8]	Observational study Bioinformatic analysis	Gene expression of ACE 2 in multiple scRNA seq datasets	From airway epithelium cell datasets: Deprez et al 2019:77,969 cells were collected by bronchoscopy at 35 distinct locations, from the nose to the 12^th^ division of the airway tree. Vieira Braga et al 2019.	Airway and Nasal epithelium	scRNA seq profiling	Spearman’ s correlation analysis with Benjamini– Hochberg.	Adjusted p values were used	Benjamini-Hochberg - adjusted p values were used. The correlation coefficient were <0.12 P value not mentioned	SARS-CoV-2 Entry receptors ACE 2 are highly expressed in nasal goblet and ciliated cells.	Higher expression of ACE 2 is seen in Nasal Goblet and ciliated cells.	Studies may lack specific cell types due to their sparsity, the challenges associated with isolation or analysis methodology. The expression may be under- detected due to technical dropout effects. Hence, to confirm the findings human studies to be carried out.
5	Descamps et al. 2020 [9]	Case control study	Human tissue biopsies from 7 different location [n=70] in the upper aero digestive tract were obtained from patients who underwent surgery in Belgian hospitals for chronic rhino sinusitis, sleep surgery or for cancer resection The kidney and small intestine tissues were used as positive and negative controls.	Oral cavity [n=10] Sinus [n=10] Vocal cord [n=10] Salivary glands [n=10] Supraglottic larynx [n=10] Tonsil [n=10] Pharynx [n=10]	Salivary glands Supraglottic larynx , Tonsil Pharynx Oral mucous membrane Sinus Vocal cord	Immunohistochemistry: The percentage of positive staining was described as 0 = no stained cells, 1 = less than 25%, 2 = between 25 to 75%, 3 = above 75%. The intensity of the staining is counted as 0 = no expression/intensity, 1 = weak intensity, 2 = moderate. 3 = strong. An immunostaining score was finally calculated by the sum of these two values (percentage +intensity) and a value from 0 to 6 was attributed to each cell type, and tissue location analyzed.	i) Mann Whitney test, ii) Spearman’ s Rho correlation test, iii) Kruskal - Wallis test	P<0.05 was considered to be significant	Quantitative analysis shows ACE 2 expression in epithelial immunostaining of 60 head and neck cases with P = 0.002	1.75% of epithelium shows strong staining for ACE 2 expression in sinus, oral mucosa, vocal cord, salivary gland. 2.25% to 75% variable staining for ACE 2 expression in tonsil and supraglottic part of laryngeal squamous epithelia. 3. Hypopharynge al epithelium shows <25% of staining. 4. Oropharyngeal epithelium showed more extensive and intense expression	High expression of ACE 2 in epithelial cells was seen in the sinus, oral mucosa, vocal cord and the salivary gland. Oropharyngeal epithelium had extensive staining for ACE 2 mainly Squamous epithelium of the Vocal cords. Weak to moderate staining for ACE 2 in the ducts and acini of salivary glands. No significant positive correlation between ACE 2 expression in epithelial cells (n=52) and the clinical parameters [Gender, age, tobacco, diabetes, arterial hypertension, blood pressure medication]	Limited sample and unmatched control size
6	Song et al. 2020 [10]	Bioinformatic analysis	GTEx database (Gene type Tissue Expression) was employed to explore the ACE 2 expression in healthy population	Bulk RNA seq profiles of 55 normal salivary gland tissues	Salivary glands	RNA seq profiling	i) Correlation analysis: Pearson’s correlation analysis, ii) Functional analysis: KEGG analysis	P <0.05 was considered to be significant	Pearson’s correlation analysis-ACE 2 expression and salivary glands were positively correlated with correlation coefficient r=0.35, p =0.01	1. Angiotensin converting enzyme 2 was expressed in salivary glands in healthy population. 2. ACE 2 expression in the salivary glands in younger population was marginally higher than in the elder population. 3. Higher expression of ACE 2 potentially activated the ribosomal pathway. KEGG analysis revealed that asthma, Spliceosome, autophagy were activated in the higher expression group.	Salivary gland shows Angiotensin Converting Enzyme 2. ACE 2 expression levels were positively correlated with TMPRSS 2 level in most organs including the salivary gland. KEGG analysis revealed increased expression of ACE 2 activated the ribosomal pathway.	No statistical significance was observed in the expression levels of ACE 2 on age disparity.
7	Hamming et al. 2004 [11]	Observational study	Human tissue specimens from 93 different subjects	Oral mucosa n=4, Nasal mucosa n=5, Nasopharynx n=6	Oral mucosa Epithelium and Endothelium of Nasal Mucosa	Immunohistochemistry	Mean age of the patients (n=93) was 52+/-22 years Male to female ratio=50:43	p value not mentioned	p value not mentioned	ACE 2 was expressed in the basal layer of non- keratinizing squamous epithelium of oral and nasal mucosa. In oral mucosa, strong staining is seen in the endothelium. Granular ACE 2 staining is seen in the basal layer of epithelium.	ACE 2 was present in endothelial cells from small to large arteries and veins. Arterial smooth muscle cells, myofibroblasts, adipose cells were positive for ACE 2. In nasal, oral mucosa and oropharynx, ACE 2 was expressed in the basal layer of non- keratinizing squamous epithelium.	Levels of ACE 2 expression in different organs were not estimated.
8	Chen et al. 2020 [12]	Observational cohort study	ACE 2 protein analysis in nasal specimens	19 biopsies of nasal tissues including olfactory epithelium, respiratory epithelial samples from chronic rhino sinusitis (CRS) patients [10 female, 9 male] 4 control patients [2 male, 2 female]	Olfactory Epithelium	Immunohistochemistry	Unpaired two- tailed Student’s t- test	P<0.05 was considered to be significant	High intensity ACE 2 staining was detected in all 13 olfactory mucosal biopsies with P =0.0001	Nasal olfactory epithelium shows increased ACE 2 expression whereas only 47.4% (9 in 19) biopsies of nasal respiratory epithelium shows ACE 2 positive staining.	ACE 2 is localized to the apical surface of sustentacular cells in the olfactory neuroepithelium. ACE 2 is also observed in Bowman’s glands and duct cells. Nasal respiratory epithelium shows significantly lower level of expression than the olfactory epithelium. In nasal respiratory epithelium, ACE 2 is located on the apical surface.	Limited sample size.
9	Sacconi et al. 2020 [13]	Observational study Bioinformatic analysis	Using databases from TCGA and the Regina Elena Institute (IRE) databases in Head and Neck Squamous Carcinoma cells	478 tumor samples and 44 normal samples from TCGA HNSCC cohort for whom both miRNA and mRNA sequencing was available. The dataset included 391HPV- and 85 HPV+ cases, with 331 p53 mutated and 147 p53 wild type cases respectively. 352 out of 478 samples were male and 126 female.	Head and Neck Squamous cell Carcinoma patients	RNA seq profiling	Two sided paired and unpaired Student’s test and ANOVA test.	p<0.05 was considered to be significant	ACE 2 Expression in tumor samples were increased with p =0.16	ACE 2 expression level in female Head and Neck Squamous Cell Carcinoma patients were up regulated.	Increased ACE 2 expression in Head and neck Squamous cell carcinoma patients and TMPRSS2 expression was significantly down regulated in Head and neck Squamous cell carcinoma patients.	As the study was based on bioinformatic analysis, expression of ACE 2 in human neoplastic tissues to be carried out to validate the findings.
10	Dai et al. 2020 [14]	Case control study Bioinformatic analysis	Starbase dataset were used to analyze the ACE 2 distribution in cancer samples	Head and neck squamous cell carcinoma samples- 502 Normal control samples-44	Head and Neck Squamous cell Carcinoma patients	RNA seq profiling	Gene expression of ACE 2 was compared between cancer specimens and controls with Student’s t-test.	P <0.05 was considered to be significant	Fold change=1.17 with p=0.47 FDR=0.62	ACE 2 expression level in Head and neck cancer patients: 1.41 ACE 2 expression level in normal samples: 1.1	ACE 2 expression in Head and Neck Squamous Cell Carcinoma patients was higher than normal controls	Unequalled d control size, To the limitation of the public data, expression of ACE 2 in human head and neck neoplastic tissues to be carried out to validate the findings.
11	Han et al. 2020 [15]	Observation al study Bioinformatic analysis	RNA seq Transcriptome expression data from GTEx (Gene type Tissue Expression) portal, Tissue Cancer Genome Atlas Portal (TCGA) from healthy subjects.	Salivary gland tissues: Case-37 Control-18 Thyroid tissues: Case-191 Control-147	Salivary gland tissues, Thyroid tissues	RNA seq profiling	The Games- Howell Test was used to compare the differences in ACE 2 expression between pairs of different organs.	P<0.05 was considered to be significant	p value not mentioned	Increased ACE 2 expression was analyzed in salivary gland tissues and thyroid tissues.	ACE 2 expressions in healthy tissues were highest in salivary gland tissues and thyroid tissues.	Expression levels of ACE 2 in different organs were not evaluated.
12	Chakladar et al. 2020 [16]	Observation al study Bioinformatic analysis	Oral epithelial solid tissue normal samples were obtained from TCGA and used to compare the gene expression between smoking and nonsmoking patients	RNA-sequencing data were obtained for 22 adjacent normal tissue samples of head and neck squamous cell carcinoma.	Oral Epithelial cells	RNA seq profiling	Kruskal–Wallis testing was performed to find associations between the expression of ACE 2 genes and the smoking status of patients.	p <0.05 was considered to be significant	p value not mentioned	ACE 2 were significantly up regulated in smokers. In oral epithelial cells, ACE 2 was up regulated is correlated to overall androgen pathway up regulation.	ACE 2 gene were significantly up regulated in oral epithelial solid tissue in smokers than non-smokers. Smoking mediated susceptibility to SARS COV 2 presented in oral epithelial cells as ACE 2 and TMPRSS 2 is up regulated in smoking patients.	Further investigation is necessary to investigate the interactions among tobacco smoke, androgens, ACE 2 including the validation of this computational data using in vitro and in vivo experiments.
13	Singh et al. 2020 [17]	Observational study Bioinformatic analysis	Public scRNA seq datasets-Bulk transcriptomics from human	Human: Ciliated cells (n=1,513), Goblet cells 2 (n=1,463) and Goblet cells 1 (n=4,017) for old samples and Multiciliated cells (n=855), Secretory cells (n=7,138) and Suprabasal cells (n=1,640) for young samples	Nasal Epithelium	scRNA seq profiling	p-value was calculated between the percentage of positive cells using one- sided Fisher’s exact test.	Adjusted P<0.01 was considered to be significant	Default cutoffs were used to identify with log FC of |0.25| and adjusted P value< 0.01.	ACE 2 RNA was most abundant in ciliated cells, ACE 2 positive cells were # 2.3% in ciliated cells# 1.6% in secretory cells # 1% in suprabasal cells	ACE 2 was most abundant in ciliated cells, nasal epithelial cells. ACE 2 factors were highly expressed in ciliated and secretory cells in higher age group than young age group.	Lack or under- representation of certain cell types that are rare or undetected due to low sequencing depth, isolation biases, or statistical cutoffs. Likewise, the expression level of any given gene may be underestimated due to dropout effects.
14	Ravioli et al. 2020 [18]	Observational study Bioinformatic analysis	From Tissue Cancer Genome Atlas Portal (TCGA) selected pan- cancer Atlas studies 35 cancer types. From GTEx portal (Gene type Tissue Expression) analyzed expression data of ACE 2 samples from 980 healthy donors.	Head and neck cancer samples n=513	Cancer patients (Oncolog ic diseases)	scRNA seq profiling	Mann Whitney U test	Adjusted P values were used	P values were adjusted for multiple testing using Benjamini-Hochberg procedure. P value not mentioned	1. ACE 2 and TMPRSS2 expressions in healthy tissues showed a higher expression in the age class 20 to 59 years (false discovery rate [FDR] < 0.0001) regardless of gender. 2. ACE 2 and TMPRSS2 were more expressed in tumors from males than females (both FDR < 0.0001) and, opposite to the regulation in tissues from healthy individuals, more expressed in elderly patients (FDR = 0.005; FDR < 0.0001, respectively). 3. Lower ACE 2 expression in head and neck cancer samples (FDR=0.005)	Elderly cancer patients had higher expression of ACE 2. ACE 2 and TMPRSS 2 expressions were higher in elderly male cancer patients when compared with healthy individuals	Unmatched control size. Results were based on systematic analysis of online public data repositories.

Interpretation of the results

Table 2 provides an interpretation of the study's findings.

**Table 2:** Interpretation of the results Results shows that angiotensin converting enzyme 2 (ACE 2) is 4.43 fold times increased in the head and neck region (OR=4.43, 95% CI, 3.76-5.22). ACE 2 expression in the head and neck tissues is 49% of that of the other tissues (OR=0.49, 95% CI, 0.05-5.05). In head and neck carcinoma patients, ACE 2 expression is 60% of that of the normal tissues (OR=0.60, 95% CI, 0.04-9.26).

Variable	Number of studies	Sample size		Odds Ratio	95% CI		Heterogeneity	
		Cases	Controls		Upper	Lower	I_2_	P value
Overall	7	19163	23295	4.43	3.76	5.22	97%	<0.00001
Head and neck region	4	183	64	0.49	0.05	5.05	74%	<0.0008
Head and neck carcinoma	3	18980	23231	0.60	0.04	9.26	99%	<0.00001

**Figure 2:**
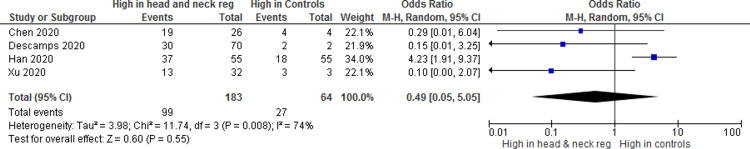
Angiotensin converting enzyme 2 in head and neck region and in controls (Xu H et al. 2020 [4], Descamps et al. 2020 [9], Chen et al. 2020 [12], Han et al. 2020 [15])

Four studies with a total of 183 patients, showed 49% of increased ACE 2 expression in the head and neck tissues than that of the other tissues (OR = 0.49, 95% CI, 0.05-5.05). Heterogeneity was evinced between the studies analyzing ACE 2 in the head and neck region: I_2_ = 74%, P_h_ ≤ 0.0008 (Figure 2).

**Figure 3:**
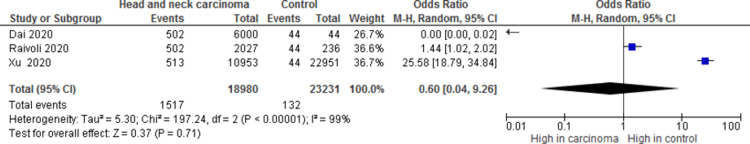
Angiotensin converting enzyme 2 expression in head and neck carcinoma cells than normal tissues (Xu J et al. 2020 [6], Dai et al. 2020 [14], Raivoli et al. 2020 [18])

In a total of three articles with 18980 head and neck carcinoma patients (10953 Oral Squamous Cell Carcinoma patients and 8027 Head and Neck Squamous Cell Carcinoma patients), the ACE 2 expression is 60% when compared to that of the normal tissues (OR = 0.60, 95% CI, 0.04-9.26). There was evidence of heterogeneity between the studies analyzing ACE 2 in head and neck carcinoma patients with I_2_ = 99% and P_h_ ≤ 0.00001 (Figure 3).

**Figure 4:**
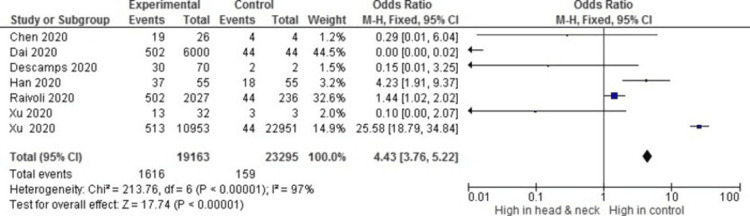
Overall analysis of studies addressing angiotensin converting enzyme 2 (ACE 2) expression is 4.43 times increasingly expressed in the head and neck region (OR=4.43, 95% CI, 3.76-5.22) (Xu H et al. 2020 [4], Xu J et al. 2020 [6], Descamps et al. 2020 [9], Chen et al. 2020 [12], Dai et al. 2020 [14], Han et al. 2020 [15], Raivoli et al. 2020 [18])

Of the total seven studies (n=7) included for the meta-analysis shows that ACE 2 is 4.43-fold times increased in the head and neck tissues (OR = 4.43, 95% CI, 3.76-5.22) with overall heterogeneity I_2_ = 97% and P_h _≤ 0.00001 (Figure 4). This evinces that ACE 2 is widely expressed all over the head and neck region, which acts as a portal for infectious disease.

Discussion

ACE 2, a counter-regulatory component of the Renin-Angiotensin System (RAS) component, was identified as the functional receptor of SARS-CoV-2. There is evidence that Renin-Angiotensin Components (RAS) are expressed locally in all the tissues. At the molecular level, ACE 2 is found in all the tissues and acts as a regulatory component of RAS. Local RAS components are not only expressed in the tissues but also participate in the initiation and progression of diseases. The most significant role of the locally acting RAS is its function at the cellular level. Their paracrine and autocrine effects are of particular importance because they mediate cell-specific effects like cell proliferation, growth, and metabolism.

Our systematic review and meta-analysis included studies analyzing ACE 2 in the head and neck region. In n = 42,458 tissue samples, a subgroup meta-analysis was performed based on the pathological condition of the tissues (normal and carcinoma tissues). Of the total 14 studies from the review, seven studies were included in the meta-analysis (Figure 2-4). The quality of the evidence for each included study was assessed using the Oxford Levels of Medicine (Oxford Centre of Evidence-Based Medicine-Levels of Evidence (March 2009)) (Table 3).

**Table 3:** Quality of evidence for each included study: Oxford Levels of Medicine ACE 2 - angiotensin converting enzyme 2; SARS CoV 2 - severe acute respiratory syndrome coronavirus 2; nCoV - novel coronavirus; TMPRSS 2 - transmembrane serine protease 2 (Xu H et al. [4], Xu J et al. [6], Sakaguchi et al. [7], Sugnak et al. [8], Descamps et al. [9], Song et al. [10], Hamming et al. [11], Chen et al. [12], Sacconi et al. [13], Dai et al. [14], Han et al. [15], Chakladar et al. [16], Singh et al. [17], Raivoli et al. [18])

S. No	Research Study	Author Year	Level of evidence
1.	High expression of ACE 2 receptor of 2019-nCoV on the epithelial cells of oral mucosa	Xu H et al. 2020 [4]	2b
2.	Digestive symptoms of COVID-19 and expression of ACE 2 indigestive tract organs	Xu J et al. 2020 [6]	3b
3.	Existence of SARS-CoV-2 Entry Molecules in the Oral Cavity	Sakaguchi et al. 2020 [7]	3b
4.	SARS-CoV-2 Entry Genes Are Most Highly Expressed in Nasal Goblet and Ciliated Cells within Human Airways	Sugnak et al. 2020 [8]	4
5.	ACE 2 protein landscape in the head and neck region: The conundrum of SARS-CoV-2 infection	Descamps et al. 2020 [9]	3b
6.	Systematic analysis of ACE 2 and TMPRSS 2 expression in salivary glands reveals underlying transmission mechanism caused by SARS-CoV-2	Song et al. 2020 [10]	4
7.	Tissue distribution of ACE 2 protein, the functional receptors for SARS coronavirus. A first step understanding SARS pathogenesis.	Hamming et al. 2004 [11]	4
8.	Elevated ACE 2 expression in the olfactory neuroepithelium: implications for anosmia and upper respiratory SARS-CoV-2 entry and replication	Chen et al. 2020 [12]	2b
9.	TMPRSS2, a SARS-CoV-2 internalization protease is downregulated in head and neck cancer patients	Sacconi et al. 2020 [13]	2b
10.	A profiling analysis on the receptor ACE 2 expression reveals the potential risk of different type of cancers vulnerable to SARS-CoV-2 infection	Dai et al. 2020 [14]	3b
11.	Analysis of 2019-nCoV receptor ACE 2 expression in different tissues and its significance study	Han et al. 2020 [15]	4
12.	Smoking-Mediated Upregulation of the Androgen Pathway Leads to Increased SARS-CoV-2 Susceptibility	Chakladar et al. 2020 [16]	4
13.	A Single-Cell RNA Expression Map of Human Coronavirus Entry Factors	Singh et al. 2020 [17]	4
14.	ACE 2 and TMPRSS 2 Potential Involvement in Genetic Susceptibility to SARS-COV-2 in Cancer Patients	Ravioli et al. 2020 [18]	3b

The overall analysis of studies addressing ACE 2 in the head and neck tissues showed that ACE 2 expression is 4.43 times more highly expressed in the head and neck region (OR = 4.43, 95% CI, 3.76-5.22) (Figure 4).

ACE 2 expression in the head and neck tissues was 49% of that of other tissues (OR = 0.49, 95% CI, 0.05-5.05) (Figure 2). The highest levels of ACE 2 expression were found in the epithelial cells of upper respiratory mucosa including the nasal cavity, ethmoid and unicate sinuses, nasopharynx, and trachea. In the sinus, oral mucosa, vocal cord, and salivary gland, 75% of epithelium show strong staining for ACE 2 [7].

Although the oropharynx and gastrointestinal tract are physiologically linked, the distribution pattern of ACE 2 varies from the oropharynx to the colon with unknown significance and mechanism. Oral epithelial and endothelial cells, small to large arteries, veins, arterial smooth muscle cells such as myofibroblasts and fibroblasts, and adipose tissue of the endothelium express ACE 2. Among the oral epithelial cells, ACE 2 levels in tongue epithelial cells were higher than in buccal and gingival tissues [4-7]. Enhanced ACE 2 expression in the olfactory epithelium suggests a mechanism of olfactory loss and a potential entry point for SARS-CoV-2. ACE 2 expressions have been detected in both the nasal and bronchial epithelium of the nasal mucosa. Goblet cells and epithelial cells lining the nasal turbinates, ethmoid sinus, and unicate sinus express the ACE 2 receptors. According to the literature, ACE 2 is primarily expressed in non-neuronal cells, as well as other cells of the olfactory epithelium. Secretory goblet cells of the nasal mucosa did not express ACE 2. ACE 2 expression is distributed in the basal layer of the non-keratinizing squamous epithelium in the nasal, oral mucosa, and nasopharynx [6]. ACE 2 expressions were demonstrated in ciliated epithelial cells, glial cells, non-neuronal cells, sustentatorial cells, and stem cells of the olfactory epithelium [8,19]. ACE 2 and transmembrane serine protease 2 (TMPRSS 2) were co-expressed by secretory cells of the nasal epithelium.

ACE 2 expressions gradually increase from the oral cavity to the esophagus, stomach, and then colon. ACE 2 positive cell ratio in digestive tract organs was significantly higher than in the lung [9]. The nuclei and cytoplasm of the spinous and basal cell layers of the oral epithelium show increased ACE 2 expression. Masticatory mucosa expresses higher levels of ACE 2 [10]. Based on the analysis in mice, ACE 2 was displayed in tongue epithelial cells in non-gustatory papillae but not in taste buds. Contrastingly, a study by Sakaguchi et al. 2020 shows that ACE 2 and TMPRSS 2 are co-expressed in the taste buds of the tongue [7]. The expression of ACE 2 in the taste buds remains unclear, although taste impairment has received particular attention as a symptom of COVID-19. Also, the literature suggests that the squamous epithelium of the vocal cords exhibited extensive staining for ACE 2 [11].

ACE 2 positive cells in salivary glands may be SARS-CoV-2 virus target cells, as RNA expression of ACE 2 genes was found to be high in human salivary glands. ACE 2 was moderately expressed by the serous, glandular cells, ducts, and acini of the salivary glands. Song et al. discovered that the level of the ACE 2 receptor is positively correlated with the level of TMPRSS 2 expression in most organs, including the salivary gland [12]. Intriguingly, higher expression of ACE 2 was recognized in the minor salivary glands than in the lung tissues [12-14].

Our subgroup analysis addressing ACE 2 expression levels in head and neck squamous cell carcinoma patients found that ACE 2 expression was at 60% (OR = 0.60, 95% CI, 0.04-9.26) (Figure 3) shows that it is elevated in carcinoma cells than normal tissues. mRNA expression of ACE 2 was higher in head and neck squamous cell carcinoma patients. ACE 2 expression levels in tumor cells were found to be higher than in normal healthy cells. ACE 2 expression is correlated with the differentiation state of epithelia [6]. Undifferentiated cells express less ACE 2 and well-differentiated cells express more ACE 2.

ACE 2 expression in tissues gradually increased from inflammation to metaplasia and then to cancer. In the tumor microenvironment, ACE 2 hydrolyzes Ang-II to Ang-(1-7), where Ang-(1-7) inhibits tumor growth. ACE 2/Ang-(1-7)/Mas axis counteracts the profibrotic effects of the ACE/Ang II/AT 1R. ACE 2 expression plays a regulating role in the signal transducer and activator of the transcription 1 (STAT 1) pathway. STAT 1 could promote the T-cell immune response and inhibit myeloid-derived suppressed cell aggregation, thus mediating an anti-tumor response. The overall expression of ACE 2 in cancer patients was comparable to that in the normal population, and ACE 2 was slightly upregulated in female and older HNSCC patients [13]. Expression of ACE 2 was positively correlated with the level of immune cell infiltration. ACE 2 positively correlates with the state of epithelial differentiation [14, 15]. ACE 2 expression with human leukocyte antigens (HLA), immune in cancer and normal tissues and found that ACE 2 expression was positively correlated with interferon-stimulated gene (ISG) in tumor samples and negatively correlated with angiogenesis and TGF-β [1]. ACE 2 was expressed directly in the immune cells of patients with head and neck cancer. Tobacco smoking induces dose-dependent effects on ACE 2 [16,17]. An experimental study by Wang Z et al. [3] shows that tobacco carcinogens cause degradation, ubiquitination of ACE 2 expression at the protein level, and upregulation of ACE 2 at the mRNA level. This discrepancy in mRNA and protein levels of ACE 2 due to tobacco usage should be investigated [18,19]. In oral epithelial tissue, ACE 2 levels were higher in smokers than in non-smokers, which could indicate the increased susceptibility of smokers to COVID-19 [19]. However, the association between tobacco carcinogens and ACE 2 expression remains controversial.

Limitations

The overall methodology quality of the studies was poor with no single study having a low risk of bias. The included studies were mostly bioinformatic analyzes (n = 10 studies) of ACE 2 using the Human Protein Atlas consortium. An important distinction between the studies is that the techniques used to detect ACE 2 differ significantly, potentially resulting in identification bias between the studies. The detection methods of ACE 2 expression used were as follows: five of the summarized studies (n = 5) used an immunohistochemistry staining technique to detect ACE 2. Ten studies (n = 10) analyzed ACE 2 expression using the RNA sequencing method; of those, four studies (n = 4) used single-cell RNA sequencing and one study (n = 1) used the bulk sequencing method for detecting ACE 2 expression. Two studies (n = 2) used the RT-PCR technique as an add-on analysis. This heterogeneity among the studies can lead to identification bias in analyzing ACE 2 expression, as some methods are more sensitive than others. In addition, the sample size taken in individual studies was limited, and statistical heterogeneity was found between the case and control groups. In the studies, there was a lack of information about demographic variables such as the gender and age of the patients. There were concerns regarding the applicability of the results of individual studies to address the review question. The findings cannot be generalized to the wider population because the studies did not include geographical diversity. Further, more studies should focus on the expression of ACE 2 in the head and neck tissues in a diverse population.

## Conclusions

Our review looks at the ACE 2 expression in the head and neck region. The meta-analysis of the studies evinced that ACE 2 is highly expressed in olfactory mucosa, nasopharynx, oral mucosa, salivary glands, and in patients with head and neck cancer. However, the currently available evidence is insufficient to conclude that ACE 2 expression is markedly present all over the head and neck tissues. This emphasizes that succeeding studies should be extended to comprehend the role of ACE 2's molecular mechanism in head and neck-specific tissues and in tumor types. A deeper understanding of the role of ACE 2 in disease and health conditions may provide novel therapeutic targets.
